# MicroRNA Profiling in Cartilage Ageing

**DOI:** 10.1155/2017/2713725

**Published:** 2017-08-14

**Authors:** Panagiotis Balaskas, Katarzyna Goljanek-Whysall, Peter Clegg, Yongxiang Fang, Andy Cremers, Pieter Emans, Tim Welting, Mandy Peffers

**Affiliations:** ^1^Institute of Ageing and Chronic Disease, William Henry Duncan Building, 6 West Derby Street, Liverpool L7 8TX, UK; ^2^Centre for Genomic Research, Institute of Integrative Biology, Biosciences Building, Crown Street, University of Liverpool, Liverpool L69 7ZB, UK; ^3^Department of Orthopaedic Surgery, Maastricht University Medical Centre, 6202 AZ Maastricht, Netherlands

## Abstract

Osteoarthritis (OA) is the most common age-related joint disorder in man. MicroRNAs (miRNA), a class of small noncoding RNAs, are potential therapeutic targets for regulating molecular mechanisms in both disease and ageing. Whilst there is an increasing amount of research on the roles of miRNAs in ageing, there has been scant research on age-related changes in miRNA in a cartilage. We undertook a microarray study on young and old human cartilages. Findings were validated in an independent cohort. Contrasts between these samples identified twenty differentially expressed miRNAs in a cartilage from old donors, derived from an OA environment which clustered based on OA severity. We identified a number of recognised and novel miRNAs changing in cartilage ageing and OA including miR-126: a potential new candidate with a role in OA pathogenesis. These analyses represent important candidates that have the potential as cartilage ageing and OA biomarkers and therapeutic targets.

## 1. Introduction

Osteoarthritis (OA) is the most common degenerative disease of joints affecting approximately 10% and 18% of men and women, respectively, over the age of 60 years [1]. It mainly affects the hands, knees, and hips with symptoms including pain, joint stiffness, and movement impairment leading to reduced quality of life [2]. The molecular mechanisms of OA though not fully understood are related to abnormal joint metabolism and an imbalance between anabolic and catabolic processes [3]. This imbalance leads to pathological changes in the joint. These present mainly thinning and progressive degradation of articular cartilage: the connective tissue that protects the joint from friction and mechanical load injury [4]. Other pathological changes include thickening of the subchondral bone, inflammation of the synovium, and formation of osteophytes [5]. Current treatments are aimed principally at relieving the symptoms rather than treating the disease. However, many patients ultimately undergo joint replacement surgery for end-stage OA. This is because the molecular mechanisms underlying this heterogeneous, age-related disease are poorly characterized.

OA is a multifactorial disease with known risk factors including genetics [6], sex, obesity, and joint injury [7]. However, the most common risk factor is age [5]. The progression and initiation of OA is facilitated by numerous stimuli and circumstances including changes in the homeostatic balance due to age. Age-related cell senescence can affect chondrocyte homeostasis and metabolism, by increasing the expression of enzymes such as matrix metalloproteinases and aggrecanases, which break down the extracellular matrix of cartilage, promoting OA development [8]. Additionally, age-related inflammation (termed inflamm-ageing) promotes the expression of cell signalling molecules, such as interleukins and other cytokines. These act as mediators of matrix degradation, contributing to OA progression [9].

MicroRNAs (miRNAs or miRs) are short (~22 nt) noncoding RNAs. They have emerged as critical cell homeostasis regulators which function through posttranscriptional modulation of gene expression by binding and repressing the expression of specific mRNA targets [10]. miRNA genes are found within intergenic or intragenic regions and are transcribed into double-stranded stem-loop structures called the primary transcript. The primary transcript is processed by the microprocessor complex, consisting of the ribonuclease DROSHA, and DiGeorge syndrome critical region 8 protein into precursor miRNAs which are exported in the cytoplasm through exportin-5 [11]. Precursor miRNAs are incorporated into the RNA-induced silencing complex. These are cleaved further by the endoribonuclease Dicer to form the single-stranded mature miRNA [12]. miRNA-mediated expression is accomplished through perfect or imperfect complementarity between the miRNA and the mRNA target. Ultimately, in animals, this leads to inhibition of translation, mRNA degradation, or both [13].

It is estimated that one-third of human genes are targeted by miRNAs [14]. This makes miRNAs potential therapeutic targets for regulating both disease and ageing molecular mechanisms. Indeed, several miRNAs have been found to play an important role in cartilage development and homeostasis, and dysregulation of specific miRNAs has been linked to OA [15–17]. This suggests miRNAs as feasible novel candidates for OA treatment targets and clinical biomarkers [18]. However, whilst there have been an increasing number of studies interrogating specific miRNAs as regulators of cartilage-specific processes in OA [19], few studies have assessed the contribution of cartilage ageing in the miRNA dysregulation evident in OA. One study found an age-related increase in miR-199a-3p and miR-193b contributing to a downregulation in collagen type II, aggrecan and SOX9, along with reduced proliferation and a reduction in miR-320c [17]. MiR-24, which regulates p16INK4a, was found to link age-related senescence and chondrocyte terminal differentiation-associated matrix remodelling in OA [20]. Furthermore, Miyaki et al. observed that miR-140 null mice developed an age-related OA-like pathology due to elevated ADAMTS5 [16].

Our previous studies have identified age-related changes in miRNAs in tendon [21], bone marrow-derived mesenchymal stem cells [22], and chondrocytes engineered from MSCs [23] and cartilage [24]. In this study, we investigated, for the first time, miRNA expression in ageing knee cartilage in order to understand further cartilage ageing and determine how this may contribute to OA. Establishing miRNAs differentially expressed in joints or cartilage during ageing and/or OA can provide basis for functional studies and potentially lead to development of novel, miRNA-based interventions against cartilage, and joint degeneration during ageing and OA.

## 2. Materials and Methods

All reagents were from Thermo Fisher Scientific, unless stated.

### 2.1. Samples

For microarray analysis, femoral intercondylar notch full-thickness cartilage from male human knees of young normal (*n* = 6; mean age ± SD 22.7 ± 4.1 years) was collected at the time of anterior cruciate ligament repair. OA cartilage was from old male (*n* = 6; 66.4 ± 15.9 years) human knees collected at the time of total knee arthroplasty. For qRT-PCR validation, an independent cohort was used which consisted of young knee cartilage from the intercondylar notch *n* = 9 (mean age ± SD 23.7 + −3.8), old “normal” cartilage from the lateral femoral condyle *n* = 5 (68.6 ± 3.8), and old OA cartilage *n* = 8 (63.1 ± 8.1) from the medial femoral condyle cartilage. All old specimens came from patients with a diagnosis of OA on preoperative knee radiographs using Kellgren and Lawrence scoring [25]. All cartilages taken were macroscopically normal. Medical ethics permission was received (Maastricht University Medical Centre approval IDs: MEC 08-4-028 and 14-4-038).

### 2.2. RNA Isolation

RNA was extracted from a cartilage once pulverised into a powder with a dismembranator (Mikro-S, Sartorius, Melsungen, Germany) under liquid nitrogen. Total RNA was extracted using the mirVana RNA isolation kit (Life Technologies, Paisley, UK) according to the manufacturer's instructions. The RNA samples were quantified using a Nanodrop spectrophotometer (NanoDrop Technologies, Wilmington, USA). The integrity of the RNA was assessed on the Agilent 2100 Bioanalyzer system using an RNA Pico chip.

### 2.3. Microarrays

600–900 ng of total RNA was labelled using the Affymetrix FlashTag Biotin HSR RNA labelling kit according to the manufacturer's instructions. Following FlashTag labelling, the biotin-labelled samples were stored at −20°C prior to hybridisation onto Affymetrix GeneChip miRNA 4.0 for 17.5 hours at 48°C 60 rpm in an Affymetrix hybridisation oven 645.

Following hybridisation, the arrays were washed using Affymetrix hybridisation wash and stain kit on the GeneChip Fluidics station 450 using fluidics script FS450_0002 and scanned using the Affymetrix GeneChip scanner 3000 7G.

### 2.4. Data Analysis

CEL files were generated using the Affymetrix GeneChip Command Console Software, and Expression Console software was used to quality control array performance. The miRNA expression data measured using Affymetrix miRNA 4.0 arrays were preprocessed using Affymetrix Expression Console with optioned method RMA for data normalisation [26]. The further statistical analyses were carried out on the 2578 miRNA probe set for *Homo sapiens* extracted from all probes and were used to determine both the detected and differentially expressed (DE) miRNAs.

The presence of each probe in the young and old samples was tested. In each test, the *p* value of the six samples was combined using Fisher's combined *p* value methods. The expression was dereplicated to a transcript level by averaging replicated probes. The *p* value associated with the presence of dereplicated expression was assigned by combining the replicated probes using Fisher's combined *p* test.

The DE analyses on the contrasting two sample conditions were performed through linear models using limma package in R environment [27]. The significance of log fold change (logFC) values for miRNAs was evaluated using *t*-tests, and the *p* values associated with logFC values were adjusted for multiple testing using the false discovery rate (FDR) approach [28]. Significantly, DE were defined as those with an FDR-adjusted *p* value <5%. Sequence data have been submitted to National Centre for Biotechnology Information Gene Expression Omnibus (NCBI GEO); E-MTAB-5715.

### 2.5. Integrated miR-mRNA Analysis and Functional Enrichment Analysis

In order to identify putative miRNA targets, bioinformatics analysis was performed by uploading DE miRNA data into the microRNA target filter module within Ingenuity Pathway Analysis software (IPA, Qiagen Redwood City, CA, USA) to produce a network of potential miRNA gene targets. Targets were then filtered on a confidence of experimentally observed or highly predictive and on the cell chondrocyte. ToppGene was used for functional enrichment analysis of the miRNA targets using ToppGene [29] with a Bonferroni FDR of less than 0.05. Biological process gene ontology (GO) terms and associated FDR values generated through ToppGene were then summarised, and the network was visualised using REViGO [30] and Cytoscape [31].

### 2.6. Real-Time Polymerase Chain Reaction (qRT-PCR)

Validation of the microarray analysis results in the dependent and independent cohorts of human knee cartilage samples was carried out using real-time quantitative PCR (qRT-PCR) analysis. Total RNA was extracted and quantified as above. cDNA was synthesized using 200 ng RNA and the miScript II RT Kit according to the manufacturer's protocol (Qiagen, Crawley, UK). qPCR mastermix was prepared using the miScript SYBR Green PCR Kit (Qiagen, Crawley, UK) and the appropriate miScript Primer Assay (Qiagen, Crawley, UK) (Supplementary file 1 available online at https://doi.org/10.1155/2017/2713725) using 1 ng/*μ*l cDNA according to the manufacturer's guidelines. Real-time PCR was undertaken using an Applied Biosystems 7300 Real-Time PCR System (Applied Biosystems, Paisley, Scotland, UK). Relative expression levels were normalised to U6 snoRNA and calculated using the 2^−ΔCt^ method [32].

### 2.7. Statistical Analysis

For statistical evaluation of qRT-PCR results, a Mann–Whitney test was performed using GraphPad Prism version 7.03 for Windows, (GraphPad Software, La Jolla California USA, https://www.graphpad.com); *p* values are indicated.

## 3. Results

### 3.1. Microarray Analysis Overview

**Figure 1:**
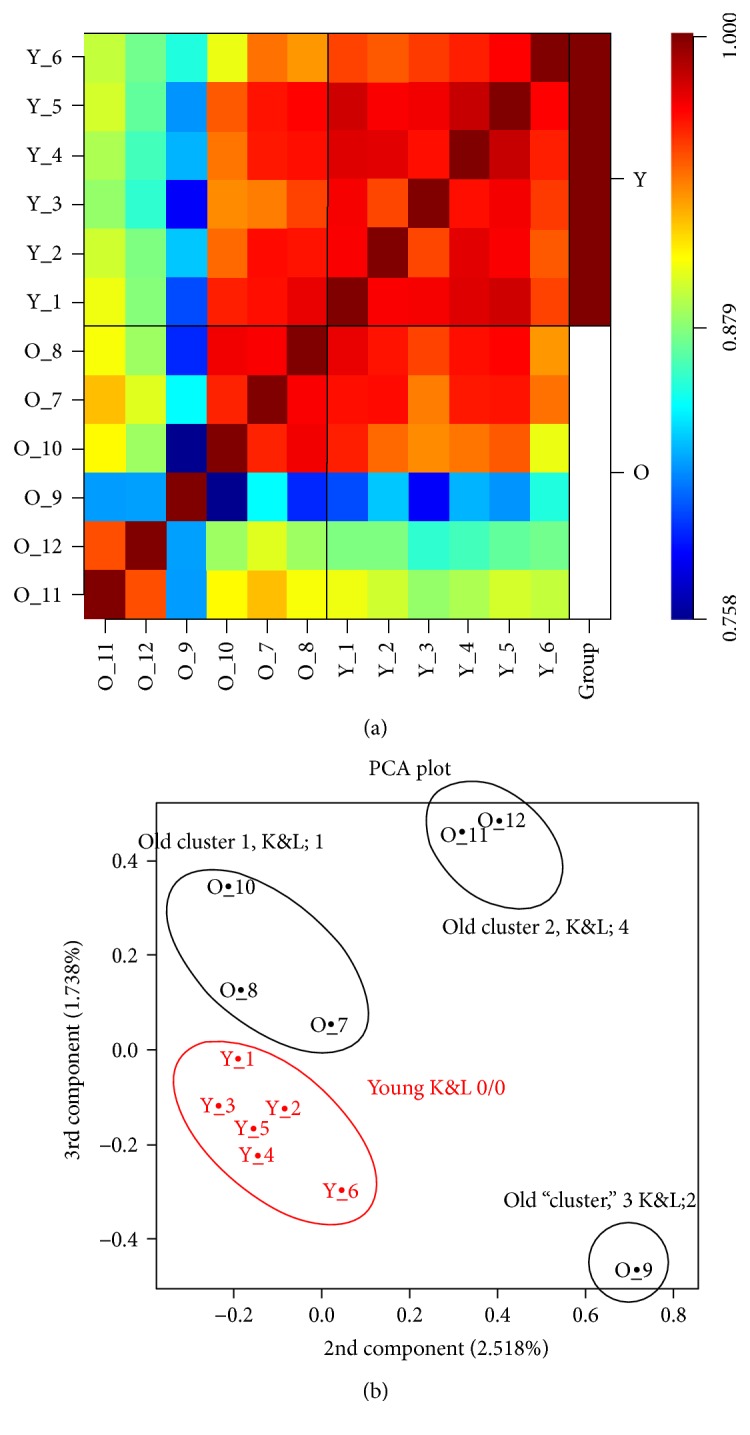
Variation data between the expressions for 12 microarray samples. (a) The heat map of hierarchical clusters of correlations among the samples. Pearson's correlation coefficients were computed using logarithm-transformed miRNA expression data from all miRNA probes that were detected. (b) A 2-D PCA plot of the second and third components from PCA of logarithm-transformed miRNA abundance data. The Kellgren and Lawrence scores (K&L) for the groups are shown on the PCA plot. H: young, O: old.

A data quality assessment report generated revealed that the quality of the data was good and consistent for all 12 arrays. The distribution for log expression signal was highly similar in signal distribution, and using a boxplot for relative log expression signal, no arrays were outliers (data not shown). The outcomes of variation assessment were visualised in Figures 1(a) and 1(b). The young samples were correlated closely together. However, the old samples were clustered into three distinct groupings as demonstrated by the correlation coefficient matrix heat map (Figure 1(a)). Principal component analysis (PCA) plot of the log expression signal for 12 arrays revealed that the samples from the young were clustered tightly together and could be separated from the old samples. However, the samples from the old group scattered in a very wide range as three subpopulations. Samples 7, 8, and 10 (cluster 1) were more similar to the samples from the young group and had the lowest KL scores: 1. Cluster 2 consisting of samples 11 and 12 had KL scores of 4 and sample 9 had KL score of 2 (Figure 1(b)). Based on the multidimensional scaling (MDS) plot, subsequently, four different selections of the old samples were made and compared to the young samples generating four result sets of DE analysis. Selection 1 includes all 6 old samples; selection 2 includes O_7, O_8, O_10, O_11, and O_12; selection 3 includes O_9, O_11, and O_12; and selection 4 includes O_7, O_8, and O_10.

### 3.2. miRNA Expression Profiling and Dysregulation

**Figure 2:**
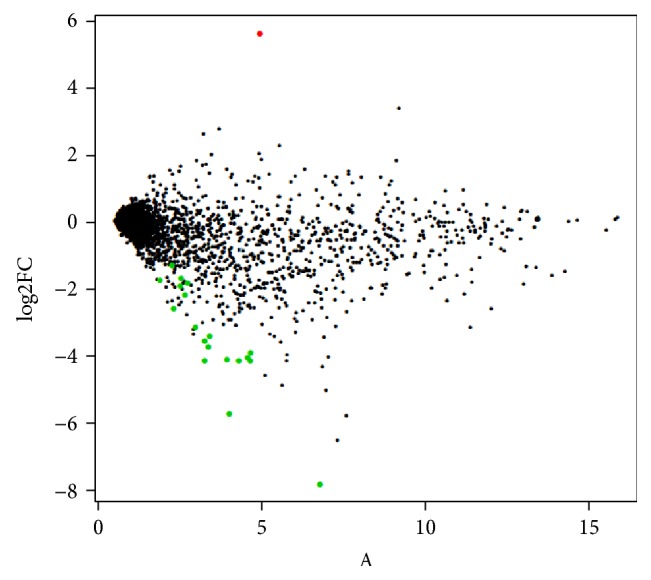
Cartilage expression profiling using an MA plot. The MA plot contrasts the log2 fold change (log2FC) against the mean intensity of all 12 arrays. The coloured spots represent DE small RNAs (FDR < 0.05), green dots reduced expression in the old OA samples, and red dots increased expression in the old OA samples. 20 miRs were significantly dysregulated; one increased in OA and 19 decreased in OA.

Of the 2578 human miRNAs represented on the Affymetrix GeneChip miRNA 4.0 microarray, 303 and 416 were detected above background in the young and old samples, respectively (Supplementary file 2). Using a cutoff of false discovery-adjusted *p* value <0.05, for selection 1 there were 20 DE miRNAs (Figure 2 and Table 1), for selection 2 there were 22 DE, for selection 3 there were 189 DE (Supplementary file 3), and for selection 4 there were 10 DE (Table 2).

### 3.3. Identification of Potential Target Genes of DE miRNAs

**Figure 3:**
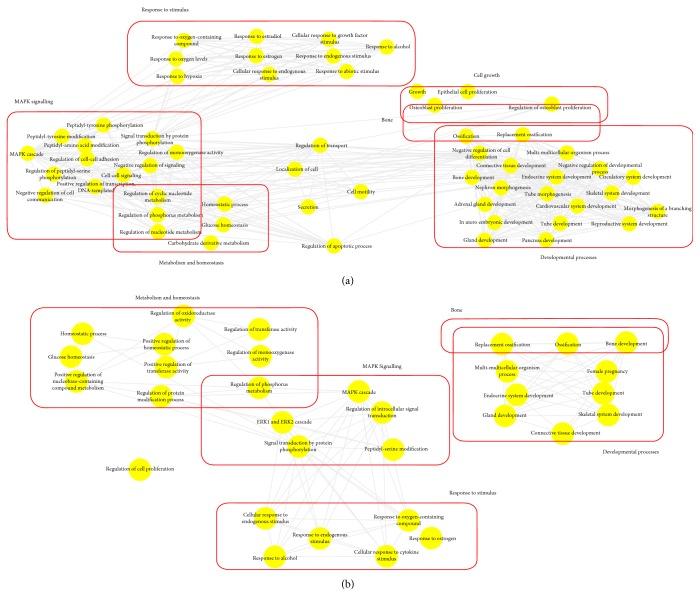
The position of the DE miRNAs in the chondrocyte expression network. Gene ontology biological processes associated with dysregulated miRNA targets were identified following TargetScan filter module in IPA. ToppGene was used to perform functional enrichment analysis on predicted miRNA targets to highlight biological processes most significantly affected by dysregulated miRNA-mRNA interactions. GO terms (FDR < 0.05) were summarized and visualised using REViGO and Cytoscape. Allowed similarity setting in Revigo was medium. The main clusters of biological processes significantly influenced by dysregulated miRs in (a) all young compared to all old samples and (b) all young samples compared to selection 4. The line width specifies the amount of similarity.

In order to investigate the position of the DE miRNAs in the chondrocyte expression network, we determined their putative target genes using IPA. This was undertaken for two datasets: (1) selection 1, the DE miRNAs were derived from all young samples compared to all old samples and (2) DE miRNAs derived from the young versus selection 4 (representing only the old samples with lowest K&L scores). This was because, we hypothesise, this latter set is most likely to be predominantly age-related changes. These presumed mRNAs were input into a gene ontology and visualised. (1) Putative target genes regulated by 11 of the 20 DE miRNAs were identified from the dysregulated genes in selection 1 (all young versus all old) in order to determine the functional significance. The microRNA target filter in IPA was used to integrate computational algorithms with multiple miRNA databases (Supplementary file 4). These presumed mRNAs were input into the gene ontology tool ToppGene, and then, biological processes were visualised in Revigo and Cytoscape (Figure 3(a)). The top biological processes were skeletal tissue development (FDR 9.29E11), regulation of cell proliferation (FDR 9.29E11), and ossification (FDR 1.18E9) (Supplementary file 3). The young samples compared to selection 4 gave putative target genes for six of the 10 DE miRNAs (Supplementary file 5). Biological processes are visualised in Figure 3(b), and the complete list is visualised in Supplementary file 5. The main biological processes were skeletal system development (FDR 3.15E07), homeostatic process (6.84E07), and positive regulation of signalling (6.22E06).

### 3.4. qRT-PCR Validation of miRNAs

To validate the changes in miRNA expression detected by microarray platform, qRT-PCR analyses using RNA from both dependent (original RNA extracted from the young normal and old OA donors used in microarray analysis) and independent cohorts were performed. An independent cohort was selected based on the samples with equivalent K&L scores to the samples used in the microarray. For the independent cohort, the K&L score from the young donors was 0, old donors mean ± SD, old “normal” 1.3 ± 0.9, and old OA 3.0 ± 0.8.

For the dependent cohort, 10 DE miRNAs from the contrast young normal versus selection 3 were selected as we decided to focus on miRNA changes due to age and OA (Table 3). The expression of miRNAs, miR-126-3p, -200c-3p, -424-3p, and -483-5p, was significantly lower in the old OA samples compared to the young normal samples (Supplementary file 6A) confirming microarray results. However, the expression of miRs, 146-5p, -424-3p, -181-5p, -let 7f-1-3p, -let 7b-5p, -150-5p, -21-5p, although following the same pattern of expression, changed between the two groups as in microarray analysis and did not reach significance (Supplementary file 6B).

**Figure 4:**
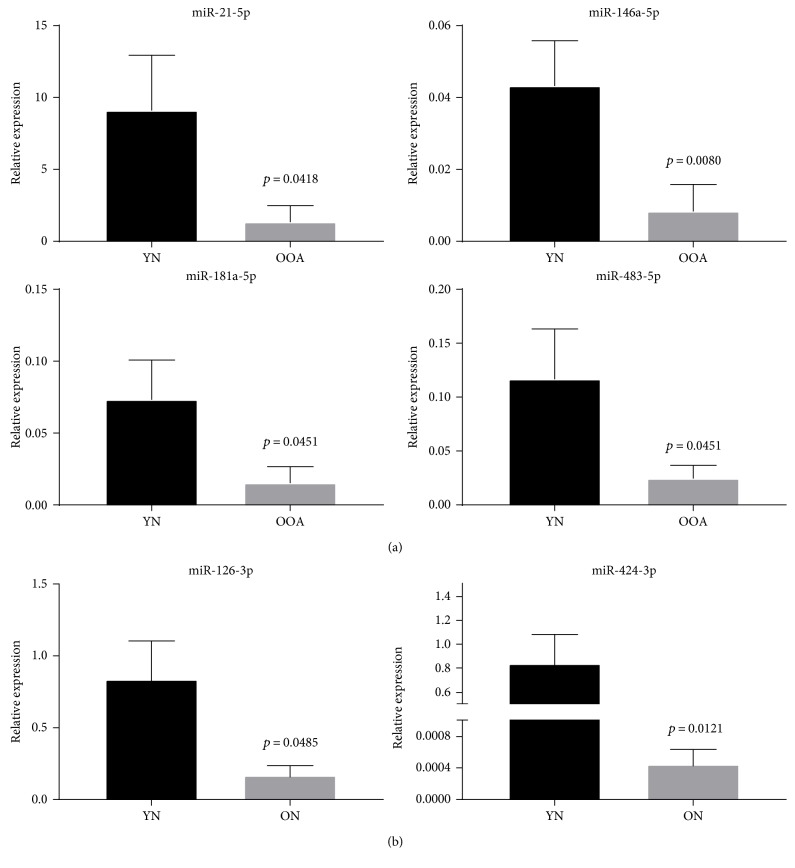
Validation of microarray results using qRT-PCR in an independent cohort. (a) Relative expression of miRNAs between the young normal and old OA cartilages. qRT-PCR results show relative expression normalised to Rnu-6 gene, young samples *n* = 7-8, old OA samples *n* = 5–7. (b) Relative expression of miRNAs between the young normal and old normal samples in an independent cohort. Results show the young normal samples (*n* = 8) and the old normal samples (*n* = 3-4). Mann–Whitney test was performed using GraphPad prism version 7.03; *p* values are indicated. YN: young normal; OOA: old OA; ON: old normal.

To further validate the results of the microarray analysis, we performed qRT-PCR analysis of the expression of miRs, -21-5p, -146a-5p, -181a-5p, and -483a-5p, on an independent cohort of samples (young normal compared to old OA). These miRNAs were chosen based on the fold change of their expression (by microarray), predicted or validated target gene set, and/or known or predicted function in cartilage maintenance and degradation. The expression of all miRNAs tested was significantly lower in the old OA samples compared to the young normal samples (Figure 4(a)). This was in agreement with the results from the dependent cohort. Additionally, in the young normal compared to the old “normal” cartilage, the miRs, -126 and -424, were validated as reduced in expression in the old normal samples in agreement with the microarray results (Figure 4(b)).

## 4. Discussion

A strong correlation exists between the age of an organism and OA, whilst ageing has a clear effect on cartilage gene expression [24]. One potential mechanism capable of regulating global alterations to a particular tissue is modification to the miRNA system. miRNAs appear to control ageing at the level of organism lifespan, tissue, and cellular senescence. The expression of many miRNAs has been demonstrated to be significantly altered with ageing. Indeed, many of these miRNAs have been identified as regulators of ageing at each of these levels. To begin to elucidate the role that miRNAs play in the global changes observed in cartilage with ageing, we undertook a microarray analysis of the young and old human cartilages. Sex-related alterations were mitigated with the use of samples from males only. We identified unique signatures which were altered with ageing and/or OA as we characterised the expression of miRNAs in knee cartilage ageing as well as DE miRNAs dependent on the severity of OA (as determined by K&L score).

In the initial microarray study, the cartilage samples were removed from the femoral intercondylar notch in both the young and old donors. This site was selected as we had access to this tissue from both donor groups. We recognise that a limitation of the study is that, although the cartilage was taken from a macroscopically normal area in the old donors, this was from an OA joint environment. Furthermore, in order to validate our microarray results, we used both the dependent cohort (to validate the platform) and an independent cohort to further validate some of our DE miRNAs in additional biological donors. The samples collected from the old donors for the latter experiment were removed from the protected (lateral; described here as old normal) or unprotected (medial; described here as old OA) condyles following TKA.

Our initial microarray analysis determined 20 DE miRNAs. However, the old donors were clustered in three groups which correlated with the severity of K&L scores. We therefore repeated the microarray analysis with each of the clusters removed. In the analysis, “selection 4,” the samples which were most different from the young (with the highest K&L scores) were removed identifying ten DE miRNAs. We hypothesize that these miRs represent the most likely dysregulated miRNAs principally due to age. These were miR-486-5p, -210, -4521, let-7a-1, -423-5p, -6795-5p, -6774-5p, -7111-5p, -6824-5p, and -6875-5p. Next, we used TargetScan to find miRNA putative target genes. Gene ontology was then undertaken on these genes in an effort to explore the position of the DE miRNAs in the chondrocyte expression network and cartilage ageing. In both the contrast between all young and all old samples, and the young versus “selection 4” (most likely affected by age or low K&L score), we identified significant biological processes. These included changes in apoptosis and cell proliferation, metabolism and homeostasis, and response to stimulus (altered nutrient sensing). Processes such as altered nutrient sensing and changes to homeostasis are some of the hallmarks of cell ageing [7]. In the young samples, compared to “selection 4,” we identified two miRNAs which are known to interact with ageing pathways. These were let-7 (cellular senescence and stem cell exhaustion) and miR-486 (altered nutrient sensing) [33]. Additionally, let-7 and miR-486 (which affect protein synthesis and mitochondrial function) have previously been identified as reduced in muscle ageing [34].

The clustering of old samples into subgroups was expected. This was as it is accepted that, although cartilage may appear grossly normal, its gene and protein expression can be affected when it is in an OA environment. Indeed, we have previously described that transcriptomes from chondrocytes in late-stage OA are similar whether cartilage is harvested from intact (protected, generally the lateral femoral condyle) or fibrillated (unprotected, generally the medial femoral condyle) areas within the knee [35]. However, others have described that in cartilage gene expression, changes are evident in different stages of OA [36, 37]. One problem with the identification of clusters apparently relating to the K&L scores was that this reduced the power of the study. Furthermore, when attempting to validate the results of the microarray with the dependent cohort using qRT-PCR, this led to large variations within the old donor groups. Higher variations in gene or miRNA expression in the old group are generally not unexpected. This is due to complexity of the ageing process, and comorbidities change occurring during ageing in the musculoskeletal system. The samples used in the old group were from two of the clusters. Thus, whilst most of the miRNAs tested with qRT-PCR showed changes in the same direction as the microarray, some did not reach statistical significance.

We believe that the analysis of the young compared to “selection 3” represents changes due to age and/or OA. These old samples represented those with the highest K&L scores compared to the young. Within this dataset, we identified 13 miRNAs known to affect the hallmarks of ageing; 11 of which were downregulated in ageing. These were for cellular senescence: let-7 and miR-146b-5p; stem cell exhaustion: let-7 and miR-29b; altered nutrient sensing: miR-120 and miR-320e; changes in gene regulation: miR-143, miR-193a, miR-200c, and miR-29b; mitochondrial dysfunction: miR-145 and miR- 349; DNA damage: miR-192, miR-24, and miR-21; inflammageing: miR-21; and loss of telomeres: miR-34a [33]. Additionally, a number of the DE miRNAs in this contrast had previously been identified in the pathogenesis of OA including miR-27b [38], miR-483 [39], miR-146 [40], miR-145 [41], and miR-675 [42]. In this study, the expression of each of these miRNAs was reduced compared to the young normal cartilage. Finally, a number of miRNAs, which have roles in cartilage homeostasis, including miR-337 [43], miR-302 [44], miR-181 [45], mir-193 [17], miR-135 [46], and miR-24 [20] were in this group. Additional work is required to decipher fully the role of this set of miRNAs in cartilage homeostasis, ageing, and OA.

Among microRNAs, DE expressed in microarray and in the dependent cohort were miRNAs, miR-126, -200c, and -424 (Supplementary file 6), whereas among miRNAs, DE expressed in microarray and independent cohort included miRNAs: -21, -146, -181 (Figure 4). MiR-483 was validated as DE expressed between the young and old OA samples in both dependent and independent cohorts. Indeed, this miRNA has been previously shown to be involved in the pathogenesis of OA [38]. It was downregulated during ageing and OA in our studies, and others have shown its positive role in cartilage maintenance [38].

Among interesting DE miRNAs in our study were miR-21, previously shown by us to be dysregulated in equine tissue during ageing [24], and also classified as “inflamma-miR” due to its major role in regulating inflammation [47]. MiR-181 demonstrated to regulate chondrocyte apoptosis in OA [48], and miR-424 previously suggested to play a role in OA [49] was also DE. Interestingly, miR-424 was also DE in the young normal compared to the old normal cohort and may also represent an age-related miR.

MiR-200c has been linked to osteogenic differentiation and proinflammatory responses by targeting interleukins 6 and 8 and chemokine (C-C motif) ligand. These are important mediators involved in OA inflammation [50]. In addition, miR-146a has been reported to play a role in cartilage homeostasis and preservation [51]. Yamasaki et al. [52] reported that expression of miR-146a was lower in late OA cartilage compared to early stages. This is in agreement with our results, where expression of miR-146a was significantly lower in the old OA donors from the dependent and independent cohorts.

Interestingly, our study has provided a new miRNA candidate, potentially regulating OA pathogenesis: miR-126. So far, little evidence exists on the role of miR-126 in joint pathology and OA. MiR-126 has been demonstrated to regulate angiogenesis and de novo vascularisation [53], as well as inflammation [54]. As cartilage is an avascular tissue, this may suggest a potential role of vascularisation, or lack of thereof in OA development. Previously, increased miR-126 expression has been described as promoting matrix-dependent cell attachment and increased cell to cell interactions between perivascular and endothelial cells during angiogenesis. Here, reduced miR-126 expression led to a less stable cell to matrix attachment network [55], in concordance with the tissue changes observed in OA. Moreover, Borgonio Cuadra et al. has reported elevated levels of miR-126 in the plasma of OA patients [56]. However, as they mention, expression levels of intra and extracellular miRNAs may differ significantly. Therefore, it is not surprising that we found reduced miR-126 expression in knee cartilage from the OA patients. Moreover, a few studies have linked miR-126 to ageing [57, 58]. Although, these studies were not relevant to cartilage homeostasis and OA, they provide indications of a possible role of miR-126 in cell ageing and senescence. Future functional studies will provide evidence on the extent to which miRNAs regulate OA development and the potential of miRNA-based interventions to ameliorate OA.

## 5. Conclusions

For the first time, we demonstrated changes in miRNAs in human knee cartilage ageing and OA. These represent miRNAs with known roles in ageing and/or OA as well as novel candidates for further functional studies. Importantly, our work provides critical evidence on the potential function of biological processes of miRNAs in cartilage ageing and OA. Further work is ongoing to determine the functional significance of specific miRNA candidates identified in this study with the aim of providing candidates as diagnostic biomarkers and therapeutic targets for OA treatment.

## Supplementary Material

Supplementary File 1. Qiagen primer assays used for the detection of mature human miRNAs through qPCR analysis. Supplementary File 2. miRNAs detected above in young and old samples. Supplementary File 3. Differentially expressed miRNAs using a cut-off of false discovery adjusted p-value <0.05 for selection 3. Supplementary File 4. Results following the use of the microRNA target filter in IPA on differentially expressed miRNAs in selection 1. Supplementary File 5. Putative target genes for miRNAs of the young samples compared to selection. Supplementary file 6: Histograms of the relative expression of miRNAs between young normal and old OA samples from the dependent cohort as measured with qPCR. A. Significantly DE miRNAs following microarray and qRT-PCR; young n=5, old n=4. B. Significantly DE miRNAS in microarray but not qRT-PCR; young n=5, old n=4.

## Figures and Tables

**Table 1:** Table demonstrating the 20 DE miRNAs in the young normal versus old OA cartilages.

miR	Log fold change	FDR adjusted
miR-126-3p	−7.81	0.01
miR-708-5p	−5.72	0.01
miR-489-3p	−4.14	0.01
miR-422a	−4.14	0.03
miR-378i	−4.14	0.03
miR-1273f	−4.10	0.01
miR-378f	−4.05	0.03
miR-150-5p	−3.91	0.02
miR-5585-3p	−3.73	0.01
miR-1273d	−3.55	0.01
miR-7111-5p	−3.41	0.01
miR-6875-5p	−3.13	0.00
miR-424-3p	−2.58	0.03
miR-6830-5p	−2.16	0.04
miR-6833-5p	−1.89	0.03
miR-6795-5p	−1.80	0.02
miR-4716-3p	−1.74	0.02
miR-4428	−1.68	0.02
miR-5010-5p	−1.29	0.02
miR-486-5p	5.64	0.00

FDR: false discovery rate.

**Table 2:** Table demonstrating the 10 DE miRNAs in the young normal versus old “selection 4” cartilages.

miR	Log fold change	FDR adjusted
hsa-miR-486-5p	5.98	0.00
hsa-mir-210	2.16	0.02
hsa-miR-4521	1.94	0.04
hsa-let-7a-1	0.93	0.04
hsa-miR-423-5p	0.82	0.02
hsa-miR-6795-5p	−1.33	0.02
hsa-miR-6774-5p	−1.42	0.04
hsa-miR-7111-5p	−2.51	0.04
hsa-miR-6824-5p	−2.76	0.03
hsa-miR-6875-5p	−2.93	0.02

FDR: false discovery rate.

**Table 3:** Summary of DE miRNAs detected by microarray analysis and selected for qRT-PCR validation.

miRNA	Expression in OOA samples compared to YN	
	Microarray analysis	qPCR analysis
let 7b-5p	**↓**	**↓**
let 7f-1-3p	**↑**	**↑**
21-5p	**↓**	**↓**
126-3p	**↓**	**↓**
146-5p	**↓**	**↓**
150-5p	**↓**	**↓**
181-5p	**↓**	**↓**
200c-3p	**↓**	**↓**
424-3p	**↓**	**↓**
483-5p	**↓**	**↓**

OOA: old osteoarthritic; YN: young normal.
