# Supplementary material for: MicroRNA Profiling in Cartilage Ageing
Source: Int J Genomics. 2017 Aug 14;2017:2713725. doi: 10.1155/2017/2713725 (PMC5584353; doi:10.1155/2017/2713725)
Supplement: Supplementary file 1 — Qiagen primer assays used for the detection of mature human miRNAs through qPCR analysis. Supplementary File 2. miRNAs detected above in young and old samples. Supplementary File 3. Differentially expressed miRNAs using a cut-off of false discovery adjusted p-value <0.05 for selection 3. Supplementary File 4. Results following the use of the microRNA target filter in IPA on differentially expressed miRNAs in selection 1. Supplementary File 5. Putative target genes for miRNAs of the young samples compared to selection. Supplementary file 6: Histograms of the relative expression of miRNAs between young normal and old OA samples from the dependent cohort as measured with qPCR. A. Significantly DE miRNAs following microarray and qRT-PCR; young n=5, old n=4. B. Significantly DE miRNAS in microarray but not qRT-PCR; young n=5, old n=4. [file 2713725.f1.docx]

| Target | Accession No | Qiagen product |
| --- | --- | --- |
| RNU6 | NR_004394.1 | Hs-RNU6_2 miScript Primer Assay |
| hsa-let-7b-5p | hsa-let-7b-5p | hsa-let-7b-5p |
| hsa-let-7f-1-3p | hsa-let-7f-1-3p | hsa-let-7f-1-3p |
| hsa-miR-21-5p | MIMAT0000076 | Hs_miR-21_2 miScript Primer Assay |
| hsa-miR-126-3p | MIMAT0000445 | Hs_miR-126_1 miScript Primer Assay |
| hsa-miR-146a-5p | MIMAT0000449 | Hs_miR-146a_1 miScript Primer Assay |
| hsa-miR-150-5p | MIMAT0000451 | Hs_miR-150_1 miScript Primer Assay |
| hsa-miR-181a-5p | MIMAT0000256 | Hs_miR-181a_2 miScript Primer Assay |
| hsa-miR-200c-3p | MIMAT0000617 | Hs_miR-200c_1 miScript Primer Assay |
| hsa-miR-424-3p | MIMAT0004749 | Hs_miR-424*_1 miScript Primer Assay |
| hsa-miR-483-5p | MIMAT0004761 | Hs_miR-483-5p_1 miScript Primer Assay |

Supplementary file 1 Qiagen primer assays for detection of mature human miRNAs through qPCR analysis.
