# Supplementary material for: MicroRNA Profiling in Cartilage Ageing
Source: Int J Genomics. 2017 Aug 14;2017:2713725. doi: 10.1155/2017/2713725 (PMC5584353; doi:10.1155/2017/2713725)

**Supplementary file 6:** Relative expression of miRNAs between young normal and old OA samples from the dependent cohort as measured with qPCR analysis. **A.** Significantly DE miRNAs following microarray and qRT-PCR; young n=5, old n=4. **B.** Significantly DE miRNAS in microarray but not qRT-PCR; young n=5, old n=4. Relative expression of miRNAs DE between young normal and old OA samples from the dependent cohort shown. Samples used for qRT-PCR were young normal (n=5) and old OA (n=4) samples from the dependent cohort. qRT-PCR results show relative expression as fold change normalised to Rnu-6. Mann-Whitney test was performed using GraphPad Prism version 7.03; p values are indicated.

A.


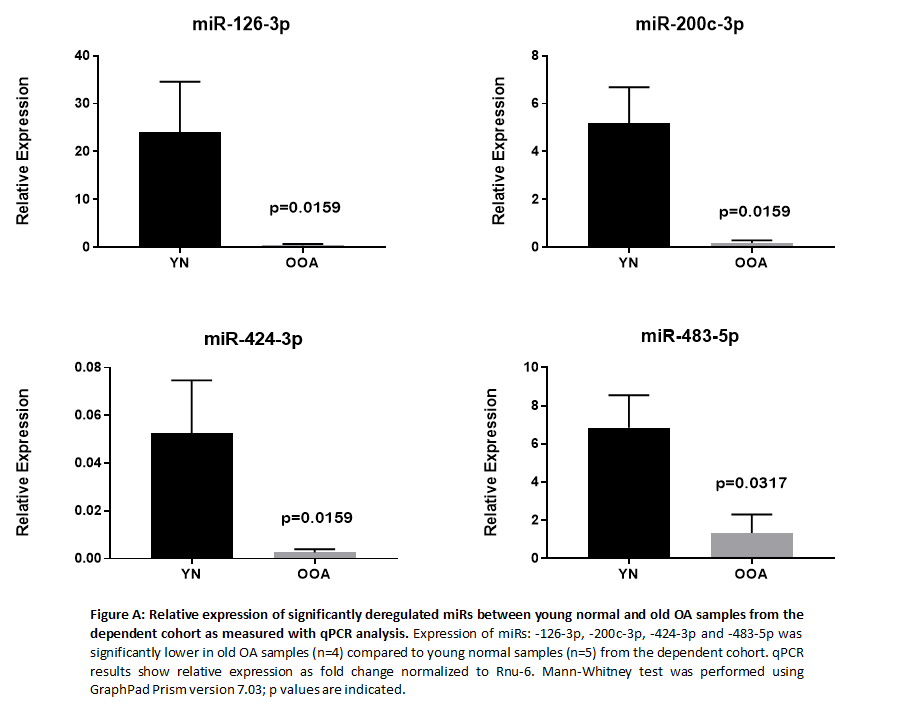


B.


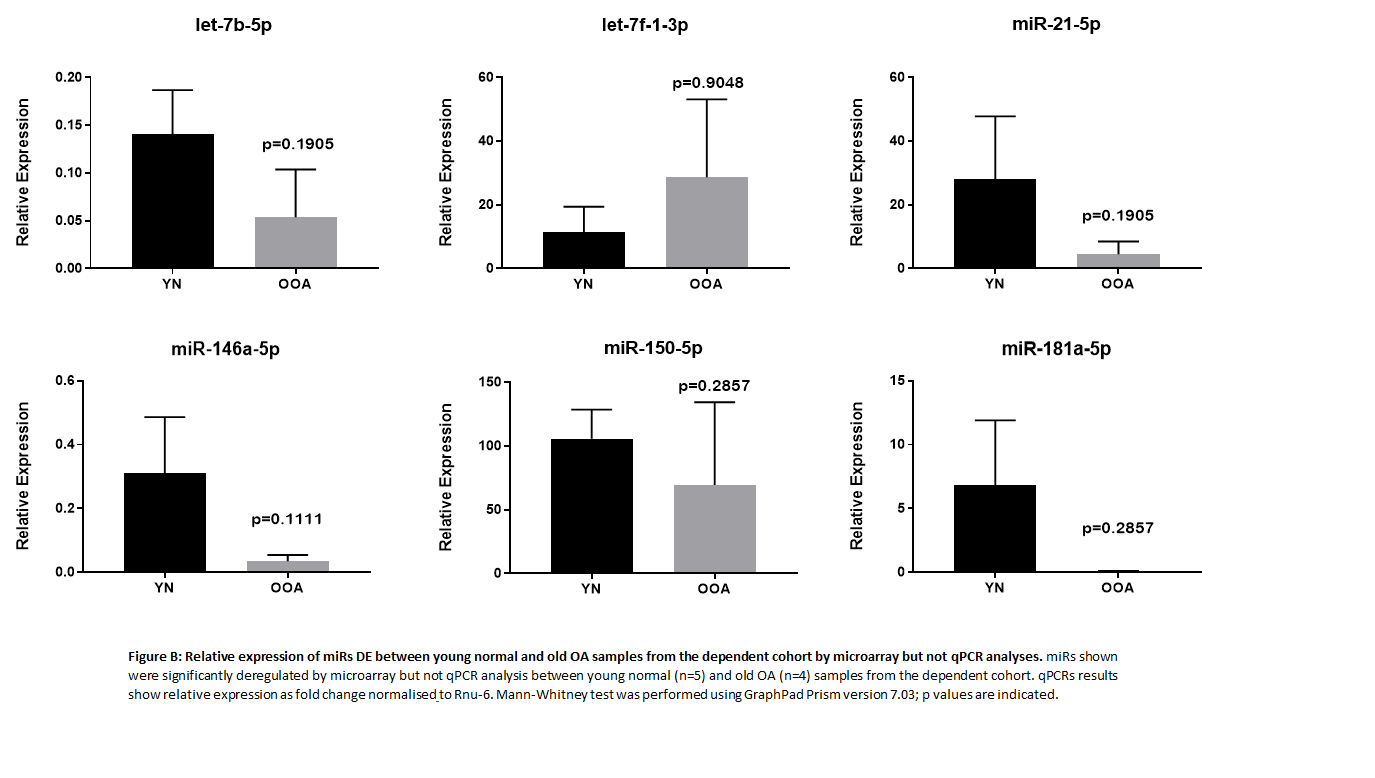

Supplement: Supplementary file 6 [file 2713725.f6.docx]
